# Targeted therapy of pyrrolo[2,3-*d*]pyrimidine antifolates in a syngeneic mouse model of high grade serous ovarian cancer and the impact on the tumor microenvironment

**DOI:** 10.1038/s41598-022-14788-5

**Published:** 2022-07-05

**Authors:** Adrianne Wallace-Povirk, Lisa Rubinsak, Agnes Malysa, Sijana H. Dzinic, Manasa Ravindra, Mathew Schneider, James Glassbrook, Carrie O’Connor, Zhanjun Hou, Seongho Kim, Jessica Back, Lisa Polin, Robert T. Morris, Aleem Gangjee, Heather Gibson, Larry H. Matherly

**Affiliations:** 1 Department of Oncology, Wayne State University School of Medicine, Detroit, MI USA; 2 Department of Pharmacology, Wayne State University School of Medicine, Detroit, MI USA; 3 Department of Biochemistry, Microbiology and Immunology, Wayne State University School of Medicine, Detroit, MI USA; 4 Barbara Ann Karmanos Cancer Institute, 4100 John R, Detroit, MI 48201 USA; 5 Division of Medicinal Chemistry, Graduate School of Pharmaceutical Sciences, Duquesne University, 600 Forbes Avenue, Pittsburgh, PA 15282 USA

**Keywords:** Cancer, Drug discovery, Immunology

## Abstract

Novel therapies are urgently needed for epithelial ovarian cancer (EOC), the most lethal gynecologic malignancy. In addition, therapies that target unique vulnerabilities in the tumor microenvironment (TME) of EOC have largely been unrealized. One strategy to achieve selective drug delivery for EOC therapy involves use of targeted antifolates via their uptake by folate receptor (FR) proteins, resulting in inhibition of essential one-carbon (C1) metabolic pathways. FRα is highly expressed in EOCs, along with the proton-coupled folate transporter (PCFT); FRβ is expressed on activated macrophages, a major infiltrating immune population in EOC. Thus, there is great potential for targeting both the tumor and the TME with agents delivered via selective transport by FRs and PCFT. In this report, we investigated the therapeutic potential of a novel cytosolic C1 6-substituted pyrrolo[2,3-*d*]pyrimidine inhibitor **AGF94**, with selectivity for uptake by FRs and PCFT and inhibition of de novo purine nucleotide biosynthesis, against a syngeneic model of ovarian cancer (BR-Luc) which recapitulates high-grade serous ovarian cancer in patients. In vitro activity of **AGF94** was extended in vivo against orthotopic BR-Luc tumors. With late-stage subcutaneous BR-Luc xenografts, **AGF94** treatment resulted in substantial anti-tumor efficacy, accompanied by significantly decreased M2-like FRβ-expressing macrophages and increased CD3+ T cells, whereas CD4+ and CD8+ T cells were unaffected. Our studies demonstrate potent anti-tumor efficacy of **AGF94** in the therapy of EOC in the context of an intact immune system, and provide a framework for targeting the immunosuppressive TME as an essential component of therapy.

## Introduction

Epithelial ovarian cancer (EOC) remains the most lethal gynecologic malignancy^1^. In 2022, approximately 19,880 women in the United States will receive a new diagnosis of ovarian cancer with 12,810 women dying from their disease^2^. This high mortality-to-incidence ratio for EOC is largely due to the development of resistance to standard cytotoxic chemotherapy in late-stage disease^3^. Additionally, a contributing factor to the high death-to-incidence rate is that approximately 75% of patients are diagnosed at an advanced stage due to the asymptomatic nature of EOC. This advanced diagnosis portends an overall worse survival as late stage presentation has a 5-year relative survival of 29%, compared with 92% for early-stage disease^3^.

Increasing attention is focusing on targeted therapies for EOC mediated through folate receptors (FRs). FRα is expressed in ~ 85% of EOCs, prompting development of FRα-targeted antibody drug conjugates (mirvetuximab soravtansine), cytotoxic folic acid drug conjugates [Vintafolide (EC145)], and targeted antifolates (ONX-0801)^4–6^. Several of these agents are in various stages of clinical development^7^. With the discovery of the proton-coupled folate transporter (PCFT; SLC46A1)^8^ and its high-level expression in solid tumors including EOC^9–12^, attention has turned to the possibility of using PCFT for therapeutic targeting. Several pyrrolo[2,3-*d*]pyrimidine antifolate inhibitors have been developed with selectivity for transport by both PCFT and FRα over the reduced folate carrier (RFC; SLC19A1), the major tissue folate transporter^10,13^.

The tumor microenvironment (TME) has emerged as a key component in disease progression and therapy resistance in high grade serous ovarian cancer (HGSOC), the most common subtype of EOC^14^. The peritoneal spread of HGSOC creates a unique TME that promotes a complex interplay between the malignant ascites and surrounding tissues. This allows for tumor progression and immune evasion^14^. The predominant innate immune cellular component in ovarian cancer-associated ascites is the tumor-associated macrophage (TAM) population, which contributes to an immunosuppressive environment^15,16^. TAMs also play an important role in metastasis and angiogenesis by releasing proangiogenic factors (e.g., vascular endothelial growth factor, matrix metalloproteinase)^17,18^. Thus, inhibiting TAMs could in principle suppress tumor progression. Interestingly, ovarian cancer-associated TAMs express FRβ, affording an opportunity to inhibit TAMs via the selective uptake of FR-targeted therapeutics^19,20^.

**Figure 1:**
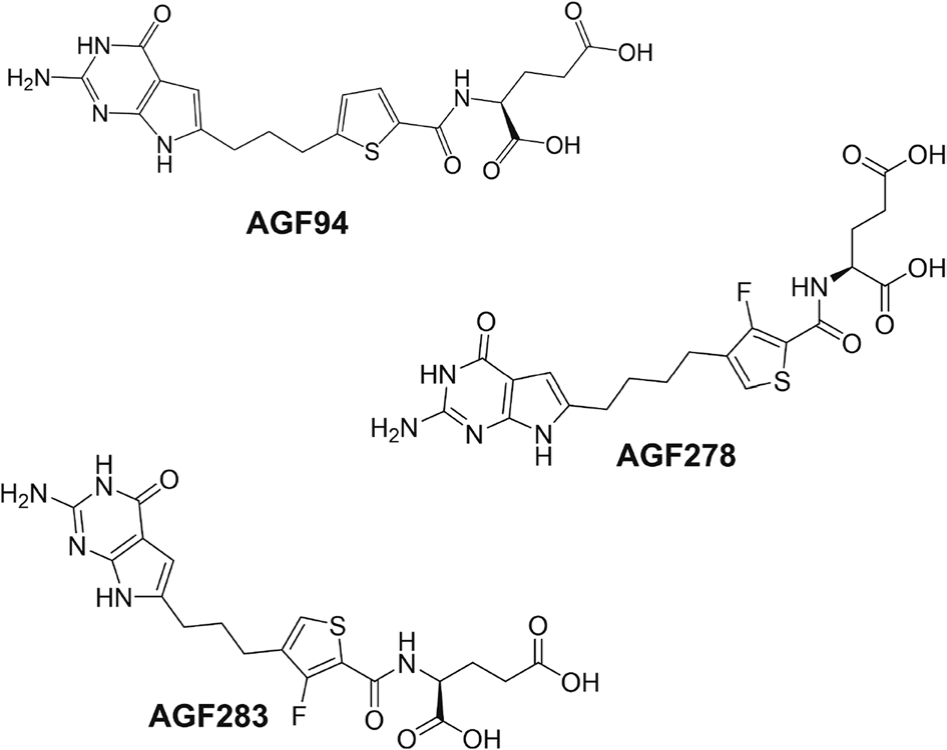
Structures of 6-substituted pyrrolo[2,3-*d*]pyrmidine inhibitors of de novo purine nucleotide biosynthesis. Structures are shown for **AGF94**, **AGF278**, and **AGF283**, selective substrates for FRs and PCFT over RFC, and inhibitors of the folate-dependent purine biosynthetic enzyme GARFTase^21,22^.

De novo purine biosynthesis is a critical pathway in tumor cells as purine depletion limits ATP and GTP for DNA synthesis and repair, and for cellular energetics. Our laboratory previously described novel 6-substituted pyrrolo[2,3-*d*]pyrimidine antifolate compounds that inhibit one-carbon (C1) metabolism in de novo purine biosynthesis, resulting in potent in vitro anti-tumor efficacy in tumor models including EOC^10,21,22^. While substantial in vivo efficacy was reported in these studies with EOC xenografts in immune-compromised mice^10,21^, anti-tumor activity in the presence of an intact immune system was not explored. Our lead analogs (i.e., **AGF94**, **AGF278**, and **AGF283**; Fig. 1) inhibited glycinamide ribonucleotide (GAR) formyltransferase (GARFTase), the first folate-dependent step in the 10 reactions comprising de novo purine biosynthesis, resulting in perturbations in purine precursors and decreased pools of purine nucleotides^10,21–23^. Anti-tumor activity of this series of compounds including EOC reflects their endocytosis by FRα and/or their facilitative uptake by PCFT^10,21^.

Reflecting the urgent need for new therapies to target this complex and highly aggressive tumor, in this report we investigated the impact of our novel FR- and PCFT-targeted pyrrolo[2,3-*d*]pyrimidine agents on both the tumor and the TME in HGSOC. To directly examine the effects of an intact immune system on the anti-tumor efficacy of our agents, we used a novel syngeneic mouse model of HGSOC (BR-Luc)^24,25^. The BR-Luc model was developed from the BR-5 EOC subline and both models are characterized by knockout of BRCA1 and p53, although BR-Luc cells also express a luciferase reporter^24,25^. Importantly, the BR-Luc EOC model recapitulates human HGSOC histology and patterns of metastasis, as well as responses to therapy^25^. We used the BR-Luc syngeneic model to investigate the relation between anti-tumor efficacy and the presence of immune infiltrates, including the impact on FRβ-expressing TAMs and CD3+, CD4+ and CD8+ T cells, accompanying treatment with the pyrrolo[2,3-*d*]pyrimidine antifolate inhibitor **AGF94**. Our results are impactful in that they further establish the translational potential of this novel series of compounds against a clinically relevant syngeneic model of HGSOC. They also provide proof-of-concept that targeting M2-like macrophages in the TME of HGSOC via FRβ likely contributes to in vivo efficacy of FR-targeted inhibitors of this class.

## Results

### Expression of folate transporters and folate-dependent enzymes in de novo purine biosynthesis in BR-5 and BR-Luc EOC cells, compared to human EOC cell lines and primary EOC and ovary specimens

We discovered novel 6-subsituted pyrrolo[2,3-*d*]pyrimidine compounds (**AGF94**, **AGF278**, **AGF283**; Fig. 1) that inhibit de novo purine biosynthesis at GARFTase and target a range of human tumor cells including EOC via selective transport by FRs and PCFT over RFC^9,10,12,21,22^. Potent in vivo efficacy by this series was demonstrated in human EOC xenografts in a severe combined immunodeficient (SCID) mouse model^10,21^. However, anti-tumor efficacy against a syngeneic mouse model of HGSOC including the potential role of the TME in treatment response has not been tested. Thus, we expanded our studies of these C1 inhibitors to include a FVB-syngeneic mouse model of EOC, BR-Luc (p53-/-; Brca-/-; myc; Akt), derived from BR-5 cells^24,25^.

We previously reported substantial expression of FRα and PCFT transcripts in HGSOC from patients^10^. FRα transcripts were significantly elevated in HGSOC over normal ovary and increased with disease stage, whereas PCFT transcripts were modestly increased over normal ovary and were independent of stage^10^. FRα and PCFT levels in EOC cell lines (i.e., SKOV3, IGROV1) were similar to those in the patient EOC specimens^10^.

**Figure 2:**
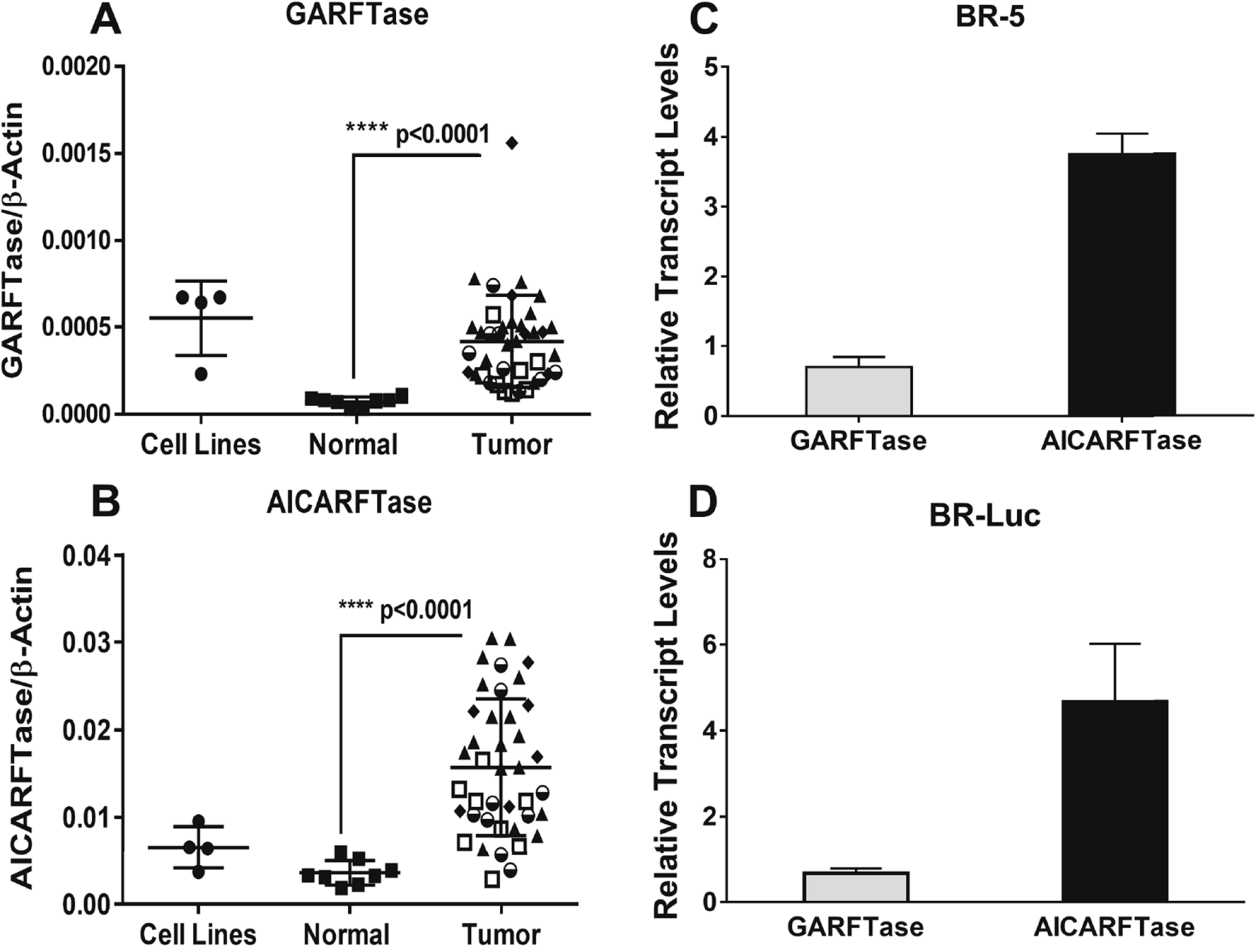
Expression of GARFTase and AICARFTase transcripts in primary EOC patient samples and BR-5 and BR-Luc cells. Transcript levels for GARFTase (**A**) and AICARFTase (**B**) were measured using cDNAs from primary specimens including normal ovary (n = 8) and HGSOC (n = 39) (OriGene) and results were compared to those for EOC cell lines including IGROV1, SKOV3, A2780 and A2780 E-80. Transcript levels were normalized to β-actin transcripts. Statistical analyses were performed between normal samples/tissues and tumor samples/tissues using the Wilcoxon rank-sum test. (**C**,**D**) Transcript levels of cytosolic C1 metabolic targets (GARFTase and AICARFTase) were determined in the BR-5 and BR-Luc syngeneic mouse models of HGSOC by real-time RT-PCR. Transcripts were normalized to β-actin transcripts and results are shown relative to levels in mouse liver (assigned a value of 1). Results are presented as mean values ± standard errors from at least three experiments. The *p* values are as follows: ****p < 0.0001. See Supplementary Table S1 for patient characteristics and pathology.

We initially analyzed gene expression of the folate-dependent de novo purine biosynthetic enzymes GARFTase and 5-aminoimidazole-4-carboxamide ribonucleotide (AICAR) formyl transferase (AICARFTase) in primary EOCs. We used a separate panel of cDNAs from before^10^, including normal ovary (n = 8) and HGSOC (n = 40; 8 stage I, 9 stage II, 17 stage III, 6 stage IV) specimens (Supplementary Table S1) to measure expression of GARFTase and AICARFTase. Transcript levels for the EOC primary specimens were compared to those in the EOC cell lines IGROV1, SKOV3, A2780 and A2780 E-80. Transcript levels for these enzyme targets in the EOC cell lines overlapped with those for primary EOC specimens; transcript levels in primary EOC specimens were substantially increased over those in normal ovary (median 5.2-fold for GARFTase; median 4.4-fold for AICARFTase) (Fig. 2A,B**)**, suggesting the importance of this key anabolic pathway to HGSOC.

For comparison, we profiled the relative expression of GARFTase and AICARFTase in the mouse models of HGSOC, BR-5 and BR-Luc. High levels of GARFTase and AICARFTase were measured in BR-5 and BR-Luc cells (equivalent to, and increased ~ fourfold, respectively, compared to mouse liver) (Fig. 2C,D).

**Figure 3:**
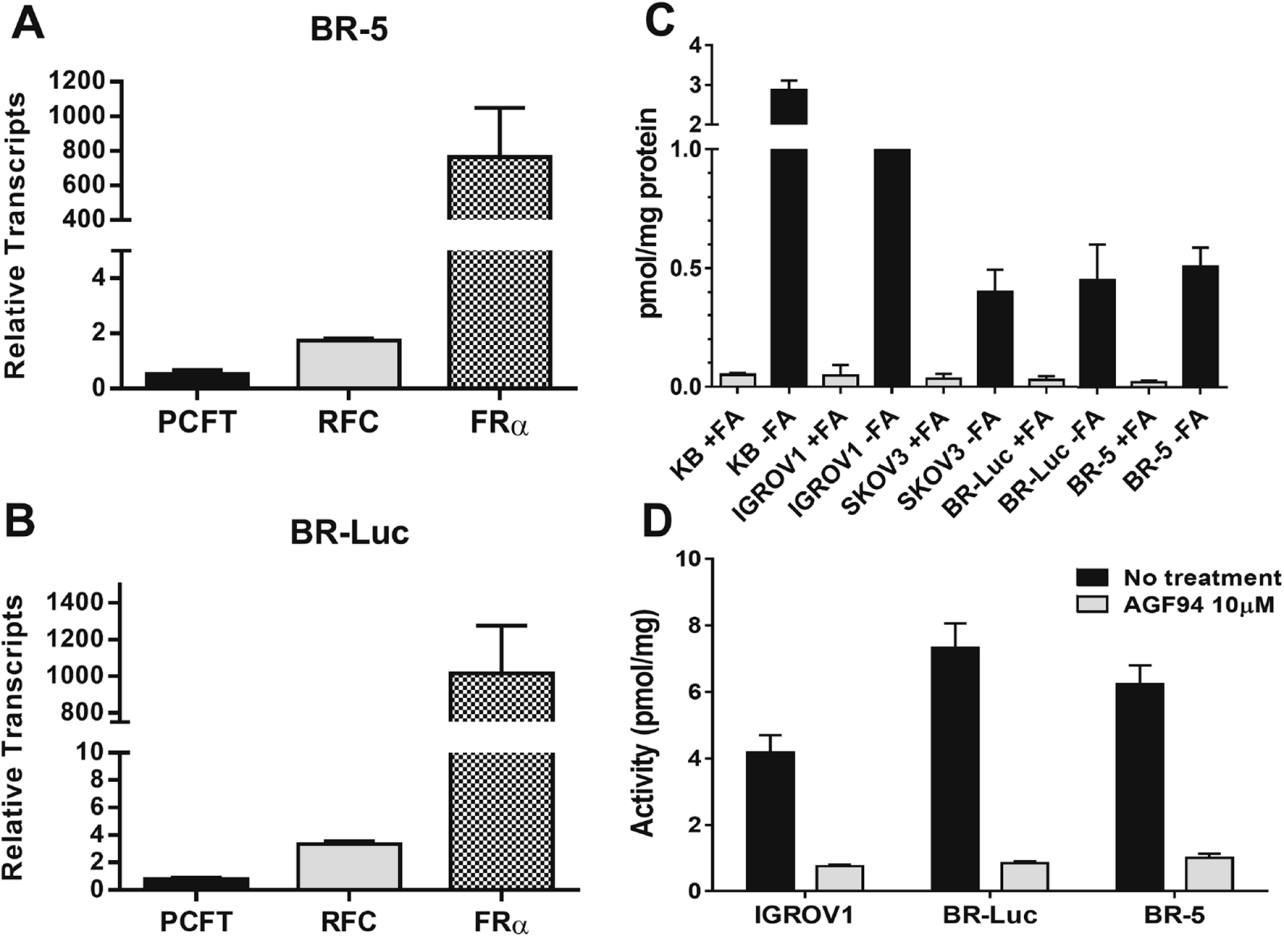
Folate transporter expression and function in BR-5 and BR-Luc syngeneic HGSOC mouse models. Transcript levels for PCFT, RFC and FRα were measured by real-time RT-PCR for BR-5 (**A**) and BR-Luc (**B**) cells. Transcripts were normalized to β-actin transcripts and levels are shown relative to those in mouse liver (assigned a value of 1). (**C**) Total surface FRα was measured by titration with [^3^H]folic acid at 0 °C with and without unlabeled 10 µmol/L non-radioactive folic acid. (**D**) PCFT uptake was assayed using [^3^H]MTX (0.5 µM) at pH 5.5 at 37 °C in the absence and presence of 10 µM non-radiolabeled **AGF94**, as previously described^22^. Results are presented as mean values (± standard errors) from at least three experiments.

We characterized BR-5 and BR-Luc murine EOC cells for expression of the major folate transporters, FRα, PCFT and RFC. The levels of PCFT and RFC transcripts in BR-5 and BR-Luc cells were substantial, with PCFT at ~ 40% and ~ 60%, respectively, of those in normal mouse liver; RFC transcripts were ~ 2- and ~ 3-fold increased over levels measured in mouse liver (Fig. 3A,B). FRα transcripts in BR-5 and BR-Luc cells were highly elevated and were increased 763- and 1015-fold, respectively, over the level of FRα in liver.

We used [^3^H]folic acid binding to surface FRs in BR-5 and BR-Luc EOC cells, as a functional readout for FRs and to compare FR protein levels in the murine EOCs to those for human tumor cell lines including FRα-expressing KB cells, and SKOV3 and IGROV1 EOC cells^10^. [^3^H]Folic acid cell surface binding was measured in the presence and absence of excess (10 µmol/L) non-radioactive folic acid to demonstrate FR binding specificity^10,26^. Total FR levels by this method were in rank order KB > IGROV1 > SKOV3 ~ BR-Luc ~ BR-5 (Fig. 3C). Thus, FRα levels in BR-5 and BR-Luc murine cells approximate those in the cisplatin resistant SKOV3 human EOC cells and by extension primary EOC specimens from patients^10^.

To functionally assess PCFT levels, PCFT transport activity was measured in the BR-5 and BR-Luc cells with 0.5 µmol/L [^3^H] methotrexate (MTX) over 5 min at pH 5.5, corresponding to the optimal pH for transport by this system^10,27,28^. Under these conditions, uptake by FRs is nominal^10^. To demonstrate specificity for transport by PCFT, we added excess (10 µM) non-radioactive **AGF94** to parallel incubations (as a competitive inhibitor) which nearly completely ablated uptake^10,21^. Results were compared to those in IGROV1 human EOC cells which express abundant PCFT along with FRα, analogous to primary patient EOCs^10^. As shown in Fig. 3D, substantial PCFT transport activity was detected in BR-5 and BR-Luc cells, with slightly increased transport in the murine EOC cells compared to human IGROV1 cells.

Collectively, these results establish that the key determinants of sensitivity to the pyrrolo[2,3-*d*]pyrimidine antifolates in the BR-5/BR-Luc murine EOC models closely approximate those of the human EOC cell lines and primary EOC specimens. These strongly suggest that, as with human EOC cells, the murine HGSOC cells should be vulnerable to the inhibitory effects of the pyrrolo[2,3-*d*]pyrimidine antifolates.

### In vitro efficacy of C1 inhibitors for BR-5 and BR-Luc murine HGSOC cells

We previously established an extensive structure–activity-relationship (SAR) profile for 6-substituted pyrrolo[2,3-*d*]pyrimidine compounds with modifications in the side chain, including the bridge length and the nature of the aromatic moiety, as selective transport substrates for FRs and PCFT, and as inhibitors of de novo purine nucleotide biosynthesis^13,27,29^. To further characterize the murine HGSOC models BR-5 and BR-Luc, we tested the in vitro antiproliferative activities of our lead pyrrolo[2,3-*d*]pyrimidine C1 inhibitors **AGF94**, **AGF278** and **AGF283**^21,22^ (Fig. 1). Results with BR-5 and BR-Luc cells were compared to those for IGROV1 human EOC cells (Table 1) in the absence and presence of excess folic acid (200 nM), which selectively blocks FR-mediated drug uptake without effects on RFC or PCFT^10^. BR-5 and BR-Luc cells showed potent inhibition by all the compounds with IC_50_ values ranging from ~ 6 nM for **AGF94** to ~ 75 nM for **AGF283** (both with BR-Luc cells) (Table 1). Further, inhibitions of the murine EOCs by all compounds approximated those for IGROV1 human EOC cells. For both murine and human EOC cells, inhibitions were significantly decreased by excess folic acid, establishing their cellular uptake by FRα with secondary uptake by PCFT^10,23^.

**Table 1:** IC_50_ values (in nM) for anti-proliferative activities of novel compounds on HGSOC mouse models BR-Luc and BR-5 compared to cisplatin.

Inhibitor	BR-Luc		BR-5		IGROV1	
	No addition	Folic acid (200 nM)	No addition	Folic acid (200 nM)	No addition	Folic acid (200 nM)
Cisplatin	290 ± 170	ND	160 ± 110	ND	857 ± 62	ND
AGF94	5.86 ± 3.67	249 ± 123	8.10 ± 4.90	346 ± 166	4.14 ± 0.94	195 ± 29
AG278	6.06 ± 3.12	588 ± 163	6.55 ± 3.24	547 ± 188	1.90 ± 0.15	426 ± 152
AG283	75.52 ± 33.28	639 ± 165	63 ± 14	598 ± 158	5.61 ± 1.20	242 ± 106

In vitro proliferation assays were performed for BR-5 and BR-Luc mouse HGSOC tumor cells (both express RFC, FRα, and PCFT). Anti-proliferative studies were performed in folate-free RPMI1640 media with 10% dialyzed FBS and 25 nM leucovorin. To measure FR-mediated drug delivery, experiments were performed in the presence or absence of 200 nM folic acid. Results are presented as IC_50_ values, corresponding to the concentrations that inhibit growth by 50% relative to cells incubated without drug. The data are presented as mean values from at least 3 experiments (± standard errors). The pyrrolo[2,3-*d*]pyrimidine antifolates were previously described^21,22^.

*ND* not done.

### In vivo analysis of efficacy for AGF94 with intraperitoneal BR-Luc EOC

An important goal was to extend our prior experience in therapeutic studies with pyrrolo[2,3-*d*]pyrimidine inhibitors which utilized subcutaneous (SC) human EOC xenografts in an immune-compromised SCID mouse model^10,22^ to evaluation of a syngeneic FVB mouse model of HGSOC. We selected the lead compound **AGF94** and chose the BR-Luc EOC model for further studies based on its in vitro sensitivity to **AGF94** which paralleled that for IGROV1 human EOC cells (Table 1). Further, the BR-Luc tumor can be imaged by luminescence detection.

As proof-of-concept of antitumor efficacy of **AGF94** in mice with intact immune function and to recapitulate the clinical-pathologic features of HGSOC, we initially used intraperitoneal (IP) engraftment of BR-Luc tumors in female FVB mice. IP presentation of the BR-Luc tumor allows for dissemination throughout the peritoneum, as occurs in patients.

**Figure 4:**
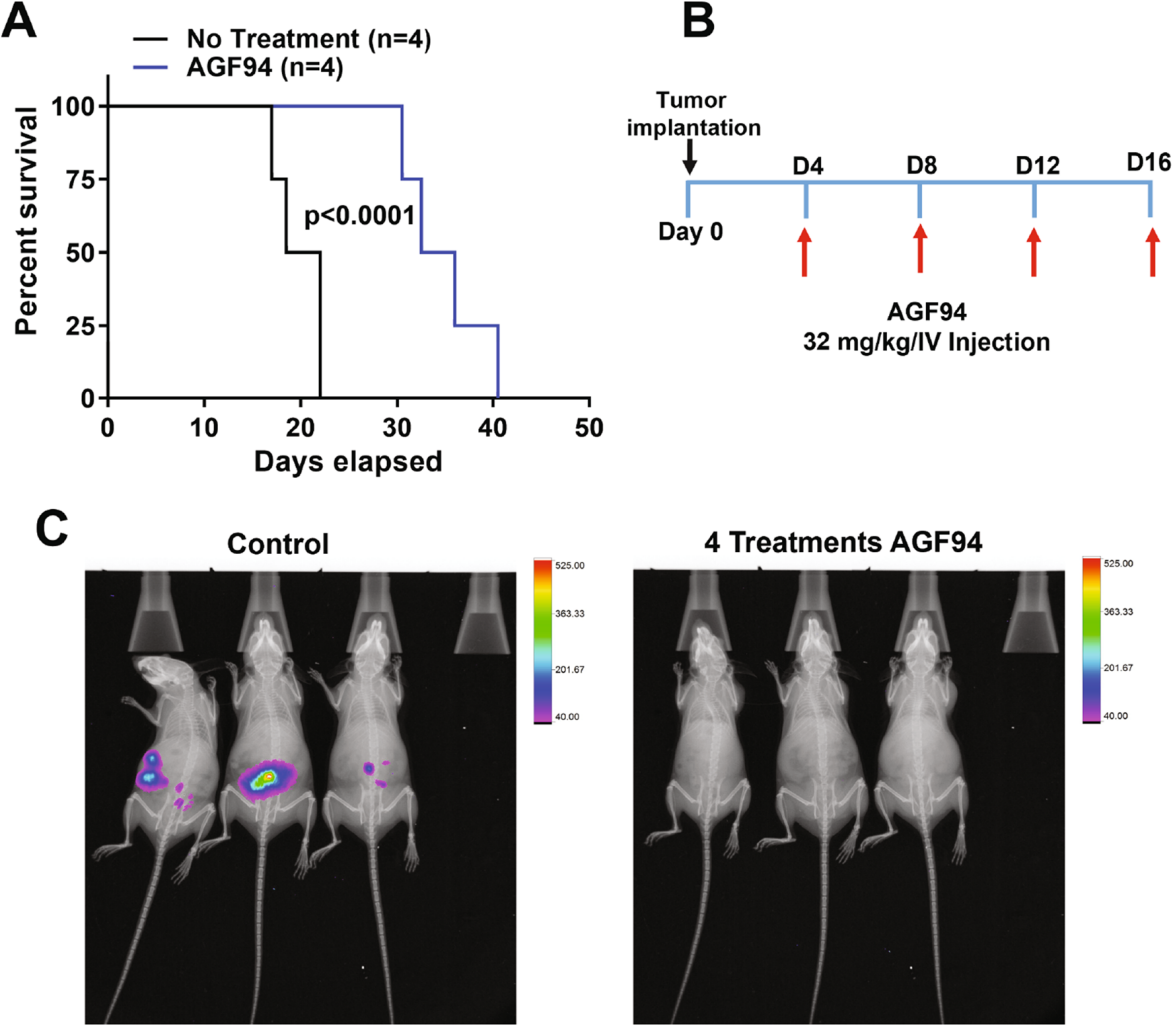
**AGF94** efficacy trial in the IP BR-Luc model. Overall survival (**A**) and tumor burden (**C**) are shown for IP BR-Luc tumors in FVB mice following treatment with **AGF94** (32 mg/kg × 4 doses). (**A**) A Kaplan–Meier survival analysis was performed on 4 mice treated with **AGF94** (32 mg/kg × 4 doses). For control mice, a median of 22 days was measured compared to a median of 33 days for the **AGF94**-treated mice. Statistical analysis was performed using the log-rank test. (**B**) The treatment scheme is shown. (**C**) Luminescent images are shown for a separate cohort (3 mice) over 3 min for control and **AGF94**-treated mice treated with 4 doses at 32 mg/kg and overlayed on top of an X-ray image.

For all in vivo studies, mice were maintained on a low-folate diet to reduce highly elevated serum folate concentrations (from the standard folate-replete diet) to levels approximating those in humans^10,21,22^. BR-Luc cells (5 × 10^6^/mouse) were injected IP on day 0, and the mice were non-selectively distributed to each arm (control and **AGF94**-treated (n = 4), with separate matching cohorts for imaging (n = 3)). **AGF94** was administered IV (Q4dx4 at 32 mg/kg/injection) beginning on day 4, for a total dose of 128 mg/kg (Fig. 4B). **AGF94**-treated mice sustained a median 5.5% body weight loss nadir on days 18 and 26 with full recovery by day 28. Disease progression and anti-tumor efficacy were monitored by weighing mice daily, palpating and measuring IP tumor masses, observing overt symptoms and luminescence imaging. Mice were euthanized at the disease end point, characterized by abdominal distension and onset of labored breathing due to ascites accumulation (> 1–2 mL), and/or the presence of palpable tumor masses > 5% of body weight (i.e., up to 1 g of cumulative solid tumor burden). Upon necropsy, accumulation of hemorrhagic ascites (1–3 mL) was observed, accompanied by disseminated metastatic disease, involving numerous small tumor nodes which localized to the ovaries, mesenteric lymph nodes and adipose tissue associated with the pancreas and GI space.

**AGF94** was active against the BR-Luc orthotopic IP model (Fig. 4A,C). Following 4 treatments with **AGF94**, tumors were undetectable by luminescence imaging (Fig. 4C shows imaging of the matched control and **AGF94**-treated cohorts including 3 mice/group), although the tumors regrew following cessation of therapy. The median days to death in the control mice were 20.25 days (range 17.5–22 days) compared to 34.25 days (range 30.5–40.5 days) for mice treated with **AGF94**. This produced a 69% increased lifespan (%ILS) and 2.1 logs of gross cell kill based on a 2-day doubling time for IP implanted cells.

### In vivo anti-tumor efficacy analysis of AGF94 against advanced-stage subcutaneous BR-Luc and impact on the infiltrating immune microenvironment

**Figure 5:**
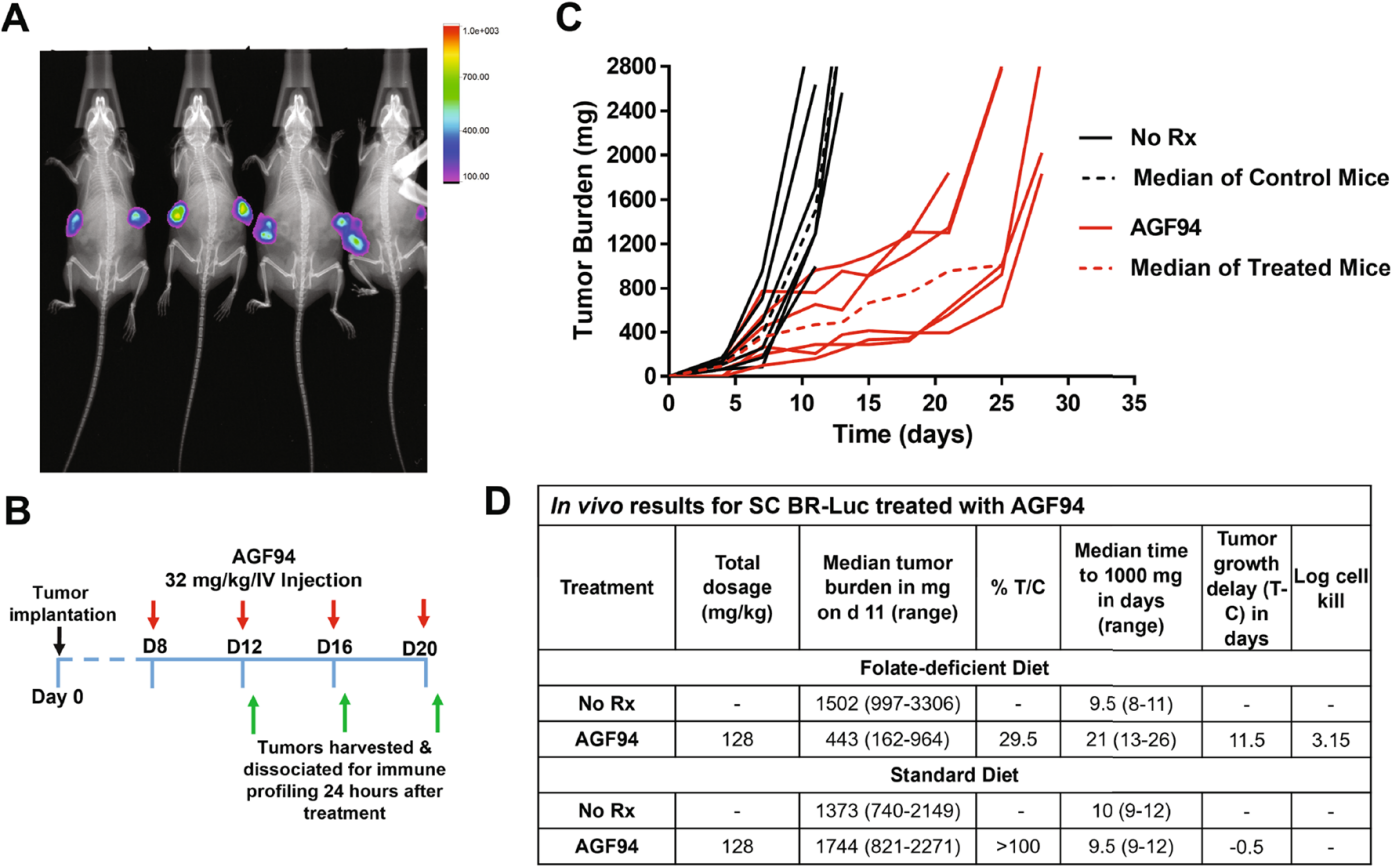
AGF94 efficacy in a subcutaneous BR-Luc model. (**A**) Luminescent images were collected over 3 min and overlayed on top of an X-ray image. The image was obtained 7 days after the tumor was allografted and 1 day prior to treatment initiation. (**B**) The trial design schematic is shown. BR-Luc tumors were engrafted SC bilaterally; treatment with **AGF94** began at 8 days when the tumors were palpable (~ 400 mg). Tumors were harvested, dissociated and flow cytometry was performed 24 h after 2, 3, and 4 doses of **AGF94**. (**C**) Results are plotted for the BR-Luc trial efficacy arm with **AGF94** by individual mice; the median results for 6 mice are shown as broken lines. Female FVB mice were implanted bilaterally SC with BR-Luc tumors and **AGF94** treatment was initiated on day 8 following tumor implantation. **AGF94** was dosed as Q4dx4 at 32 mg/kg/IV injection. %T/C values were determined on day 11. (**D**) The table summarizes the results of the in vivo trial with SC BR-Luc xenografts treated with **AGF94** for mice maintained on both the folate-deficient and standard folate replete diets.

We extended our initial in vivo studies with **AGF94** to advanced-stage SC implanted BR-Luc tumors in FVB mice which permits careful monitoring of the relationships between anti-tumor efficacy and the impact of drug treatment on the infiltrating immune microenvironment without considerations of metastatic spread. FVB mice were maintained on a folate-deficient diet and were implanted bilaterally SC with BR-Luc tumor fragments by trocar on day 0 (Fig. 5B). The tumors were measured on day 7 and the mice were then non-selectively distributed into treatment and control groups (6 mice/group) for determinations of antitumor efficacy, with parallel cohorts to accommodate imaging (n = 4, 2 each for control and **AGF94**-treated groups) and analysis of tumor immune infiltration (6–9 each for control and **AGF94**-treated groups). The median tumor burden for BR-Luc advanced stage disease was 388 mg (range 356–412 mg; day 7). Matching control and treated cohorts (n = 4 mice per group) maintained on a standard (folate replete) mouse diet had a median tumor burden of 218 mg (range 213–223 mg; day 7). The mice were dosed with **AGF94** (32 mg/kg IV on a Q4dx4 schedule), starting on day 8. Figure 5B summarizes the treatment scheme and Fig. 5A shows the luminescence detection of BR-Luc tumors on day 7 before initiation of **AGF94** treatment. All mice were weighed daily for the duration of study, as well as observed and assessed prior to each treatment or imaging. Tumor growth was monitored two-to-three times weekly until the tumor burden endpoint (5–10% of body weight) was reached.

For mice maintained on the folate-deficient diet, anti-tumor activity 3 days post treatment with **AGF94** (day 11) was reflected in a %T/C value of 29.5% (median) (Fig. 5D; further determinations of %T/C were not possible due to euthanization of controls on or after day 11, reflecting a doubling time (Td) of 1.1 days for the SC BR-Luc tumor). For quantitative assessments of anti-tumor efficacy, tumor growth delay (T–C) was used (defined as the median time for the tumor to reach 1 g for treated (T) and control (C) groups). **AGF94**-treated mice yielded a median 11.5 day tumor growth delay and 3.15 logs of gross cell kill (defined as T–C/3.32 × Td) (Fig. 5C,D). Again, **AGF94** treatment was well tolerated with the only dose-limiting symptom being weight loss (nadir of 6.4% on day 18 and full recovery by day 26). For the mice maintained on the standard (folate-replete) diet, **AGF94** was inactive (%T/C > 100%, Fig. 5D; i.e., there was no weight loss or other adverse symptoms observed).

An important goal of our study was to explore the impact of 2, 3 or 4 doses of **AGF94** upon the tumor microenvironment, including infiltrating lymphocytes accompanying its anti-tumor effects. Further, since **AGF94** is transported in part by FRs, including FRβ^22^, we measured its effects on the FRβ-expressing TAMs based on published reports that targeting macrophages via FRβ may have therapeutic potential for treating inflammatory diseases and cancer^19,20,30^.

**Figure 6:**
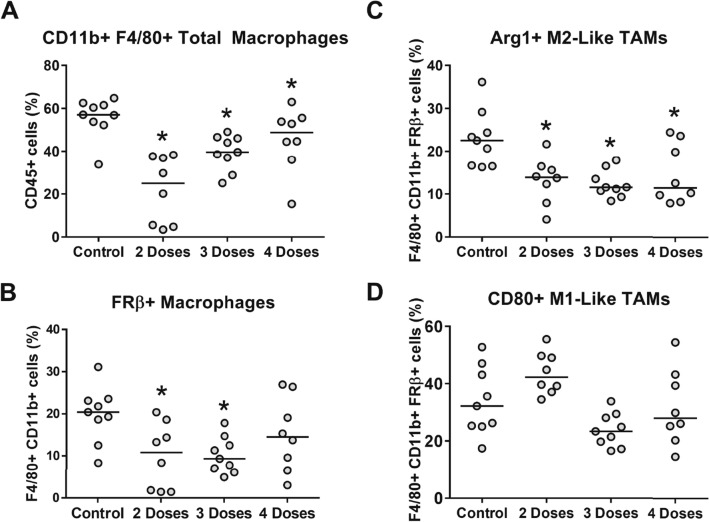
Impact of AGF94 treatment on tumor infiltrating macrophages. To assess the impact on the immune populations in mice treated with **AGF94**, the immune populations from tumors and spleens were harvested after 2, 3, and 4 treatments. (**A**) Results are shown for the percentage of CD11b+ and F4/80+ macrophages. (**B**) Percentage of FRβ+, gated off CD11b+ and F4/80+ macrophages, are shown with 2, 3, or 4 doses of **AGF94**. (**C**) Percentage of Arg1+ FRβ+, gated off CD11b+ and F4/80+ macrophages, are shown following 2, 3 and 4 doses of **AGF94**. (**D**) Percentages of CD80+ FRβ+, gated off CD11b+ and F4/80+ macrophages, are shown following 2, 3, and 4 doses of **AGF94**. Results are shown for individual mice. Horizontal bars represent median values. Statistical comparisons were made between **AGF94**-treated and control groups. *p < 0.05.

To assess the effects of drug treatment upon the infiltrating immune population, tumors and spleens (as a control) were harvested from parallel cohorts of **AGF94**-treated mice after 2, 3 and 4 injections, as well as from untreated control mice. The impact of drug treatment was initially determined on the total macrophage population, defined as dual expressing CD11b+ and F4/80+ cells from live CD45+ cells including the FRβ-expressing population (Fig. 6A,B). We measured the FRβ-expressing CD11b+/F4/80+ cells with FRβ-specific antibody (Supplementary Materials, Figure S1). After treatment with **AGF94**, there were statistically significant decreases after 2 and 3 doses on the CD11b+/F4/80+/FRβ+ population which appeared to diminish with subsequent dosing compared to untreated mice.

We extended our analysis to include the M1-like macrophage population defined as FRβ+, CD80+ TAMs, and the M2-like macrophage population defined as FRβ+, Arg1+ TAMs^31,32^. **AGF94** exerted at most a modest impact on the M1-like TAMs from 2 to 4 doses (Fig. 6D); however, there were consistent and highly significant decreases in the M2-like TAMs (Fig. 6C) that generally paralleled the observed changes in the CD11b+/F4/80+/FRβ TAM population (Fig. 6B). Thus, **AGF94** appears to target M2-like TAMs that express FRβ^20^.

**Figure 7:**
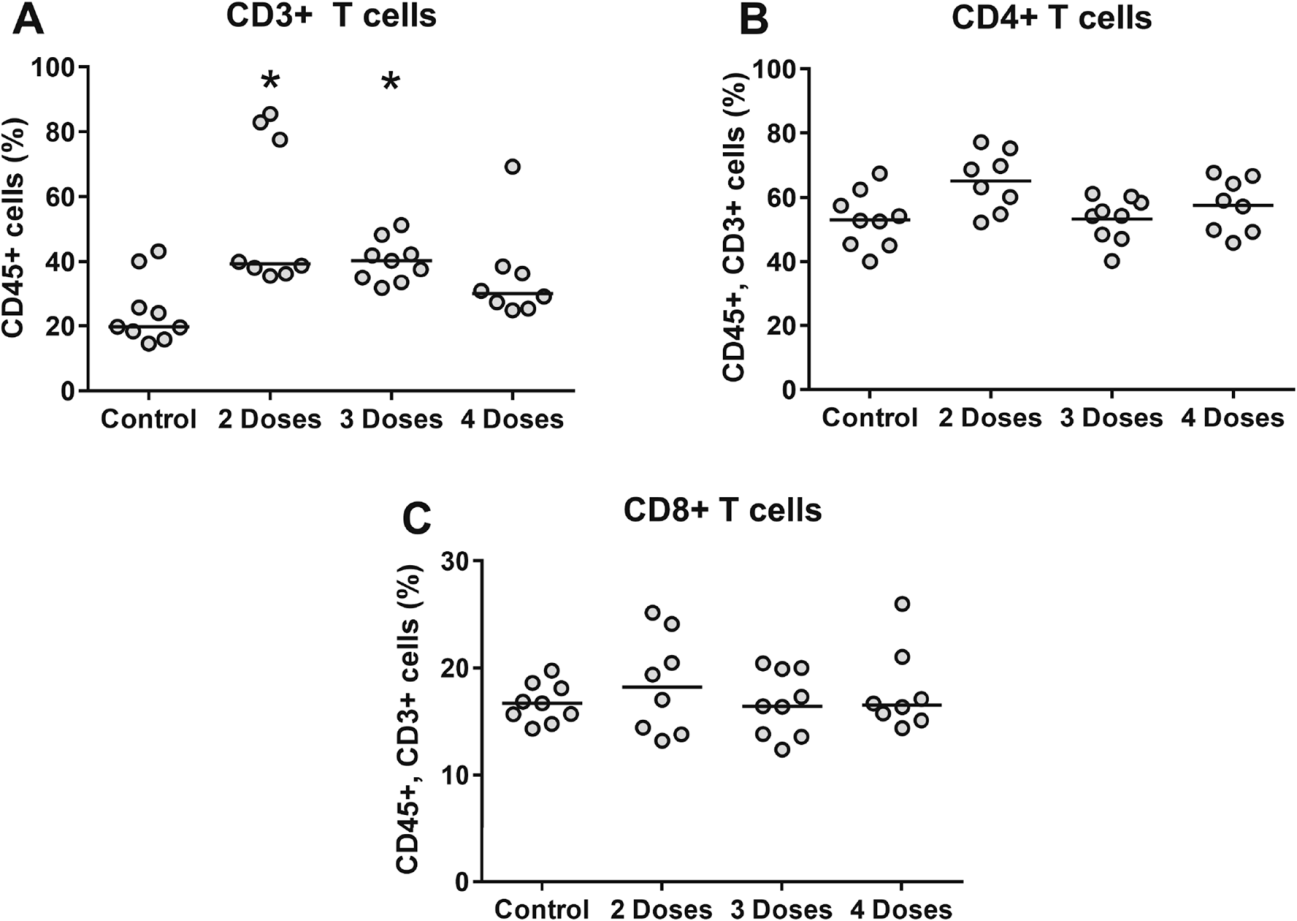
Impact of AGF94 treatment on tumor infiltrating T cells. Percentages of CD3+ T cells (**A**) are shown from single cell suspensions of BRLuc tumor and infiltrating lymphocytes following 2, 3 and 4 doses of **AGF94**. The CD3+ population was gated off CD45+ cells. Percentage of CD4+ (**B**) and CD8+ (**C**) T cells are shown from single cell suspensions of tumor and infiltrating lymphocytes following 2, 3 and 4 doses of **AGF94**. The CD4+ and CD8+ populations were gated off CD45+ and CD3+ cells. Results are shown for individual mice. Horizontal bars represent median values. Statistical comparisons were made between **AGF94**-treated and control groups. *p < 0.05.

We also examined the impact of **AGF94** dosing on the infiltrating T cell populations. Treatment with **AGF94** was accompanied by a statistically significant increase (~ 50–100%) in the CD3+ T cells, as defined by the percentage of live CD45+ cells (Fig. 7A). There was no impact on the proportions of CD4+ and CD8+ T cells in the tumors (Fig. 7B,C) and spleens (not shown) (measured as a percentage of CD3+ T cells) after 2, 3, or 4 doses of **AGF94**.

**Figure 8:**
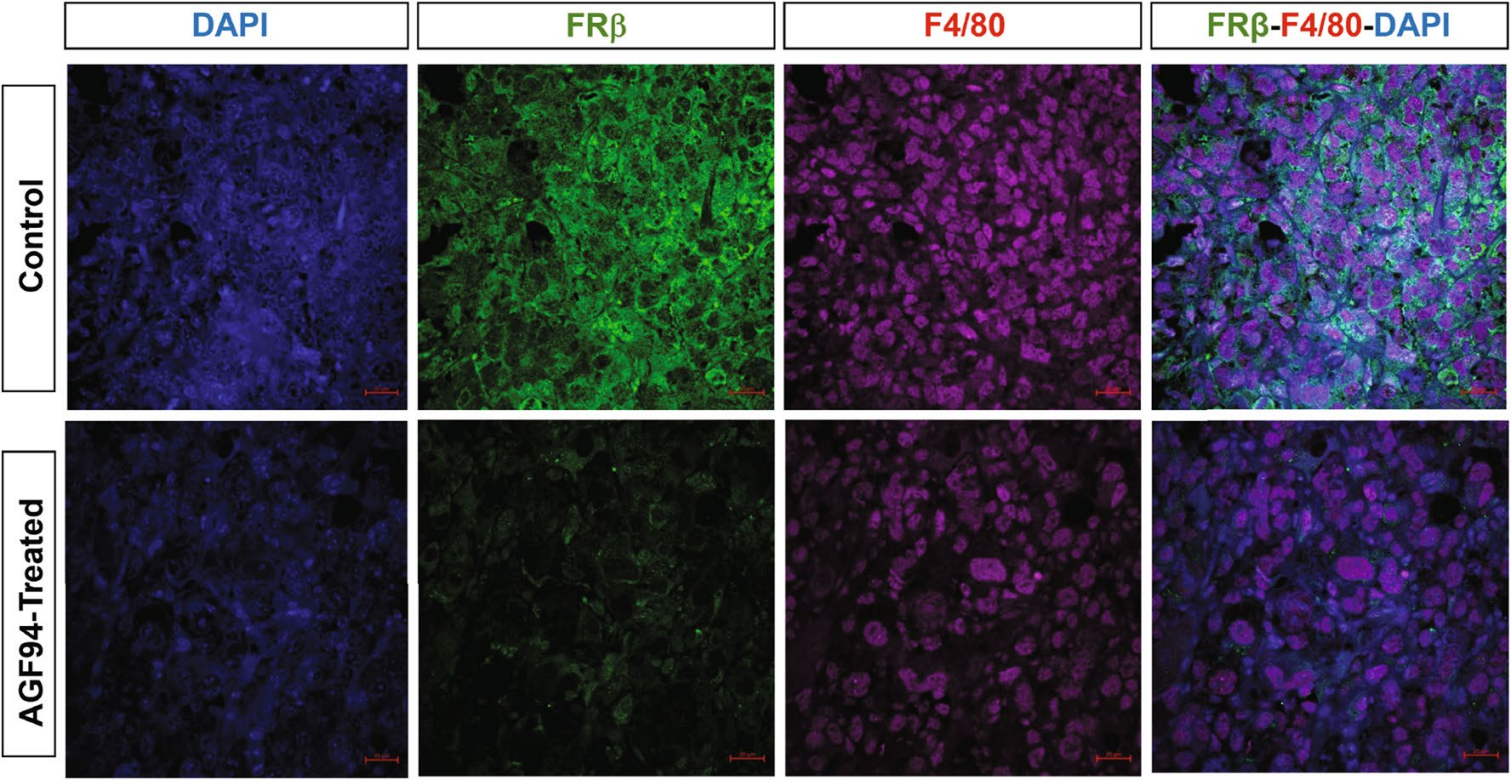
Immunofluorescence staining of FRβ-expressing tumor-associated macrophage population following treatment with AGF94. Slides were stained with FRβ (Genetex) with Alexa Fluor 488 secondary antibody and pan-Macrophage, F4/80, conjugated to APC-R700 (BD Biosciences). Images were acquired by confocal microscopy. Immunofluorescence staining is shown for representative tumor sections from control mice and mice treated with 4 doses of **AGF94**. The scale is 20 µm.

As validation of our flow cytometry results and to assess the spatial landscape of the immune infiltrate, we performed immunofluorescence (IF) staining on tumor sections from control and drug-treated mice following 4 doses of **AGF94**. Both CD3+ and CD8+ T cells were detected in sections from **AGF94**-treated mice, albeit to variable extents, consistent with the flow cytometry results (Supplementary Materials, Figure S2). Importantly, T cells were disseminated throughout the tumor bed regardless of treatment status, substantiating the quantitative flow cytometry results which suggest that these cells are not diminished by treatment with **AGF94**. Dual staining was performed with FRβ antibody and pan-macrophage F4/80+ antibody. As expected, FRß positive cells were most easily detected in the sections from untreated mice, with substantially fewer FRß positive cells in the **AGF94**-treated sections (Fig. 8).

## Discussion

The tumor microenvironment contains a host of infiltrating immune cells, including TAMs and T-lymphocytes, with TAMs considered the principal immune cellular component which results in an immunosuppressive environment^15,16^. FRβ is expressed on IL-10-producing M2-like macrophages (CD163+, CD68+, CD14+ IL-10), corresponding to the anti-inflammatory/pro-tumor TAM subtype, prompting substantial interest in depleting TAMs by exploiting FRβ on the surface of macrophages^19^. A BIM (BCL-2-interacting mediator of cell death) plasmid encapsulated in a folate “lipoplex” was developed to target the tumor microenvironment in lung cancer^30^ and an anti-mouse FRβ monoclonal antibody conjugated to *Pseudomonas* exotoxin A depleted TAMs and reduced tumor growth a C6 rat glioma model^33^. Further, a folate-conjugated TLR7 agonist showed in vivo activity in assorted tumor models and reversed expression of a high M2-like to M1-like macrophage ratio and increased the infiltration of cytotoxic CD8 T cells^20^.

The present study explored the potential of the novel pyrrolo[2,3-*d*]pyrimidine antifolate **AGF94** for dual targeting HGSOC directly, as well as indirectly via its effects on the tumor microenvironment. **AGF94** is a prototype FR- and PCFT-targeted pyrrolo[2,3-*d*]pyrimidine antifolate previously reported to show broad-ranging anti-tumor efficacy (including human EOC xenograft models)^9,10,12,21^. **AGF94** is potent inhibitor of GARFTase^22,34^, the first folate-dependent step in de novo purine biosynthesis, a critical anabolic pathway in malignant cells^35^. Inhibition of purine nucleotide biosynthesis kills tumors independent of the wild-type/mutant p53 status^36,37^, shows tumor selectivity based on impaired purine salvage^38,39^, and results in suppression of mTOR signaling^40,41^.

We used a syngeneic immunocompetent FVB mouse model of orthotopic HGSOC that accurately recapitulates the histology and progression of human HGSOC^24,25^ and found that **AGF94** effected potent in vivo anti-tumor efficacy. Our results with the orthotopic IP BR-Luc EOC model were extended to an advanced stage SC model of BR-Luc so as to better study the changes in the TME immune populations accompanying treatments with **AGF94**. Anti-tumor activity was accompanied by a direct impact on the TME including significantly decreased FRβ-expressing TAMs, and no evidence of CD3+ T cell depletion or impact on the relative proportions of CD4+ and CD8+ T cells after drug treatment.

Based on these results, we suggest that an FRβ-transported C1 inhibitor such as **AGF94** represents an exciting new approach for therapy of HGSOC through its ability to directly target the tumor via uptake by FRα and PCFT, and its effects on the TME, particularly FRβ-expressing TAMs. Of course, our results also raise important new questions that will be the focus of future studies. For instance, what are the relative contributions in targeting tumors with **AGF94** via FRα and PCFT versus FRβ-expressing TAMs to the net anti-tumor response observed, and are the CD3+ T-cells functional after treatment? As PCFT was reported to be expressed with FRβ in M2-like macrophages^42^, is there an additional role for PCFT in the uptake of our targeted agents in the pro-tumor M-2 like subtype of TAMs. While FRβ-targeting may be important for decreasing TAMs in response to **AGF94** treatment, the role of C1 metabolism in the inflammatory response is still evolving. Indeed, C1 metabolism could represent in a unique vulnerability for TAMs as serine metabolism was associated with an inflammatory response in macrophages via its impact on glutathione synthesis^43^ and S-adenosyl methionine depletion was reported to inhibit inflammatory macrophages^44^. Of course, the impact of purine inhibitors such as **AGF94** may have a broader impact, via effects on adenosine pools in the tumor microenvironment from tumor cells which act as an immune modulator resulting in macrophage polarization^45^ and inhibition of cytotoxic effector functions of NK and CD8+ T cells^46–48^. Future studies will explore the general impact of treating HGSOG with **AGF94** and related agents on C1 metabolism in relation to direct targeting tumor cells vis á vis inflammatory macrophages and T-cells in therapeutic response.

## Materials and methods

All methods were performed in accordance with the relevant guidelines and regulations.

### Reagents

All chemicals were obtained in the highest available purities from commercial sources. Leucovorin [(6R,S) 5-formyl-THF] and MTX were provided by the National Cancer Institute (Bethesda, MD). [^3^H]MTX (10–30 Ci/mmol) and [^3^H]folic acid (32.9 Ci/mmol) were purchased from Moravek Biochemicals (Brea, CA). Cisplatin was purchased from Tocris Bioscience (Bristol, UK). The novel pyrrolo[2,3-*d*]pyrimidine antifolates **AGF94**, **AGF278** and **AGF283** were synthesized as previously described^21,22^. Additional chemicals were purchased from commercial sources in the highest available purities.

### EOC cell lines and antiproliferative experiments

BR-5 and BR-Luc murine EOC cells were generous gifts from Dr. Sandra Orsulic (UCLA)^25,49^. KB nasopharyngeal carcinoma and SKOV3 cells were obtained from the American Type Culture Collection (Manassas, VA). IGROV1 (NCI-IGROV1) (passage 5) clear cell carcinoma cells were obtained from the Division of Cancer Treatment and Diagnosis, National Cancer Institute (Frederick, MD). A2780 and A2780-E80 cells were generous gifts from Dr. Gen-Sheng Wu (Karmanos Cancer Institute, Detroit, MI). All the cell lines were cultured at 37 °C under 5% CO_2_ in complete folate-free RPMI 1640 supplemented with 10% fetal bovine serum (FBS) (Sigma-Aldrich; St. Louis, MO) and 100 units/mL penicillin/100 μg/mL streptomycin, and 2 mM *L*-glutamine. All cell lines were authenticated by STR analysis by Genetica DNA Laboratories (Burlington, NC) and tested for *Mycoplasma* by PCR using a Mycloplasma testing kit (Venor™ GeM Mycoplasma Detection Kit, Sigma). Frozen stocks were generated from authenticated mycoplasma-free cultures.

Cell proliferation assays were performed as described^10,26^. Cells were plated in 96-well dishes at densities ranging from 2500 to 5000 cells/well in 200 μL media and treated with a range of inhibitors spanning concentrations from 0 to 1000 nM. Experiments used BR-5 and BR-Luc cells and folate-free RPMI 1640 media with 10% dialyzed FBS and 100 units/mL penicillin/100 μg/mL streptomycin, supplemented with 25 nM leucovorin and 2 mM *L*-glutamine. FR-mediated drug uptake was assessed in parallel incubations including 200 nM folic acid. Cells were treated over a 96 h period at 37 °C with 5% CO_2_. Relative cell numbers were quantified using the CellTiter-blue cell viability assay (Promega, Madison, WI) and a fluorescence plate reader^26^. Raw data were exported to Excel for analysis and the results plotted using Graphpad Prism 6.0. Determinations of IC_50_s were made corresponding to the drug concentrations that resulted in 50% loss of cell growth.

### Real-time RT-PCR of transcripts for the major folate transporters and C1 metabolic targets

RNAs were isolated from the murine (BR-5, BR-Luc) and human (IGROV1, SKOV3, A2780 E-80, and A2780) EOC cell lines using TRIzol reagent (Life Technologies). cDNAs were synthesized with random hexamers and MuLV reverse transcriptase (including RNase inhibitor; Applied Biosystems), and purified using a QIAquick PCR Purification Kit (Qiagen).

Human cDNAs were purchased from Origene (HORT502) containing 48 lyophilized cDNAs from EOC patient specimens (8 stage I, 9 stage II, 17 stage III, and 6 stage IV) and 8 cDNAs from normal ovaries. Patient pathology characteristics are in Supplemental Table S1.

Quantitative real-time RT-PCR was performed using a Roche LightCycler 480 real-time PCR (Roche Diagnostics) with gene-specific primers for mouse PCFT, RFC, GARFTase, AICARFTase, and FRα, or human GARFTase and AICARFTase, as appropriate (Supplemental Table S2), and FastStart DNA Master SYBR Green I Reaction Mix (Roche Diagnostics, Indianapolis, IN). Transcript levels were normalized to β-actin transcripts. For the murine transcripts, levels were normalized to transcript levels in mouse liver.

### [^3^H]Folic acid binding as a measure of surface FRs

Total FRα levels were measured for the KB, IGROV1, SKOV3, BR-5, and BR-Luc cells with a functional readout involving measuring [^3^H]folic acid binding to surface FRs^10^. Cells were plated at a density of 1–2 × 10^6^ cells in complete folate-free RPMI1640 (10% FBS) media. Cells were allowed to adhere to the plates for 24 h. The following day, the cells were washed (3x) with 4 °C Dulbecco’s phosphate-buffered saline (DPBS). Cells were washed with acetate buffer (10 mM sodium acetate, 150 mM NaCl, pH 3.5) (3 ×) at 4 °C to release FRα-bound folates, then neutralized with Hepes-buffered saline (HBS) (20 mM Hepes, 140 mM NaCl, 5 mM KCl, 2 nM MgCl_2_, and 5 mM glucose, pH 7.4) at 4 °C. The cells were incubated at 0 °C for 15 min with [^3^H]folic acid (50 nM, specific activity 0.5 Ci/mmol), in the presence or absence of non-radioactive folic acid as a competitor (10 µmol/L). After a 15 min incubation, the cells were washed with ice-cold HBS and proteins were solubilized with 0.5 N NaOH. Cell homogenates were assayed for radioactivity with a liquid scintillation counter and protein concentrations were measured using the Folin-phenol reagent^50^. Levels of [^3^H]folic acid bound to FRs were expressed as pmol [^3^H]folic acid/mg protein.

### PCFT transport assays

PCFT transport assays were performed with IGROV1, BR-5 and BR-Luc cells. Cells were plated in 60 mm dishes containing complete folate-free RPMI 1640 with 10% FBS, including 2 mM *L*-glutamine, and antibiotics; cultures were used when they were 80–90% confluent. Uptake of [^3^H]MTX (at 0.5 µM) was measured over 5 min at 37° C in MES-buffered saline (20 mM MES, 140 mM NaCl, 5 mM KCl, 2 mM MgCl_2_, and 5 mM glucose; pH 5.5). Under these conditions, uptake by FRs is negligible. To quench transport fluxes, dishes were washed 3 times with ice-cold DPBS. Cells were solubilized in 0.5 N NaOH and radioactive contents and protein concentrations^50^ were determined. Uptake was expressed as pmol [^3^H]MTX per mg protein. To confirm PCFT-mediated transport activity, 10 µM nonradioactive **AGF94** was added to the transport incubations to block PCFT uptake.

### In vivo studies

The mouse studies were approved by the Wayne State University Institutional Animal Care and Use Committee (IACUC). Syngeneic female FVB mice were purchased from Charles River Labs (Wilmington, MA) or Envigo (Indianapolis, IN). In vivo tumor maintenance and drug efficacy studies for SC BR-Luc tumors are completely analogous to those previously described^9,10,21,22^.

For the in vivo therapeutic trials, study mice were maintained on either a folate-deficient diet from HarlanTeklad (Envigo, Indianapolis, IN) (catalog # TD.00434) or a folate-replete diet from Lab Diet (catalog # 5021; autoclavable mouse breeder diet) (for both SC and intraperitoneal (IP) trials) starting 9–14 days before tumor implant (depending on tumor staging). Mice were supplied with food and water ad libitum. Serum folate levels were determined prior to tumor implant and post study via *Lactobacillus casei* bioassays^51^.

In vivo studies using the BR-Luc tumor were performed using both IP and SC presentations depending upon the desired study objective. The BR-Luc tumor was first established subcutaneously from cultured cells with implanted donor mice used to set up the efficacy studies. BR-Luc tumors from donor mice were aseptically harvested, mechanically dissociated into single cell suspensions, centrifuged, and suspended in sterile chilled saline and injected IP into study mice (5 × 10^6^/0.2 mL per mouse) on day 0. The mice were unselectively distributed into control and **AGF94**-treated groups (4 mice/group for efficacy; a separate cohort of 3 mice/group was used for imaging). Treatment (IV tail vein; 0.2 mL volume) with **AGF94** was initiated 4 days post-tumor implantation at 32 mg/kg injection on a Q4dx4 schedule (total dose of 128 mg/kg*).* The mice were weighed and observed daily for symptoms from drug treatment and disease onset (abdominal distention, palpable internal masses), with periodic monitoring of internal progression by bioluminescent imaging. To evaluate the qualitive efficacy of **AGF94** treatment, mice were imaged 24 h after receiving 2, 3, and 4 doses of drug. Imaging was performed with a Bruker CareStream in vivo Xtreme (Billerica, MA). Mice were injected IP with 150 mg/kg of XenoLight D-luciferin bioluminescent substrate (Perkin Elmer; catalog #122799). Imaging was initiated 10 min after D-luciferin injection after anesthetization with isoflurane with 2% oxygen (Fluriso; VetOne) (3% for induction and 1.5% for maintenance). For the IP study, mice were euthanized at defined disease endpoints (> 2 mL ascites, internal cumulative tumor mass up to 1 g or at onset of lethargy or respiratory impairment).

For the SC efficacy trial, mice were implanted bilaterally along the flanks via sterile 12-gauge trocar with 30 mg tumor fragments aseptically harvested from tumor donor mice on day 0. Tumors were measured every 3–4 days. On day 7, when tumor burdens approached 350–450 mg (by caliper; representative of advanced stage disease), the mice were non-selectively distributed into control and treatment groups (n = 6/group), with parallel cohorts included for imaging (n = 2/group) and analysis of immune infiltration (6–9 mice/group). Luminescent imaging was performed on day 7 to establish a baseline for advanced disease. Treatment with **AGF94** was initiated on day 8 on a Q4dx4 schedule at 32 mg/kg IV injection (days 8, 12, 16, 20). Mice were weighed and observed daily and tumors measured by caliper 2–3 times weekly, with weekly luminescent imaging for parallel cohorts. All mice were euthanized at harvest or study endpoint when the tumor burden reached 5–10% of the body weight. Tumor volumes (in mg) were calculated using the formula, length × width^2^/2 (ascertained from caliper measurements in mm, where mg = mm^3^). Total tumor burden per mouse (in mg) was determined by addition of tumor volumes on the right and left flanks. Median tumor burden was determined on each measurement day for each group. These values were used in the efficacy analysis (below).

Quantitative endpoints for the IP study include tumor growth delay (T–C) using the median survival time (median time of sacrifice) in days for treated (T) and control (C) groups, and the percent increase in lifespan (% ILS) using the formula T–C/C × 100. Log cell kill calculations were performed as described for the SC study below. Tumor volume doubling times (Td) were determined by best fit straight line from log-linear growth plot of control group tumors in log growth phase (100–800 mg for SC tumors) or by the difference in median survival times in days of titered no treatment controls (i.e., 10^6^ and 10^4^ cells, using the formula Td = median survival in days for 10^6^ − median survival time for 10^4^/3.32 × 2).

Quantitative endpoints for the SC study include: (1) tumor growth delay [T–C, where T is the median time in days required for the treatment group tumors to reach a predetermined size (e.g., 1000 mg), and C is the median time in days for the control group tumors to reach the same size; tumor-free survivors are excluded from these calculations]; (2) gross log_10_ cell kill (LCK), determined by the formula LCK = (T–C; tumor growth delay in days)/3.32 × Td (tumor doubling time in days determined by growth plot); and (3) T/C values (in percent), corresponding to periodic caliper measurements using the median tumor burden for treatment (T) and control (C) groups when control tumors are still in exponential growth phase (i.e., 500–1250 mg).

### Tumor and spleen dissociation and flow cytometry analysis of immune populations

After 2, 3, and 4 treatments of **AGF94** for the SC treatment trial, mice from the respective study arms were euthanized and tumors and spleens were dissociated. Tumors were dissociated using a GentleMACS dissociator (Miltenyi Biotec, Bergisch Gladbach, Germany) and the manufacturer’s protocol for mouse tumor dissociation. Following dissociation, tumors and associated immune infiltrate cells were resuspended with 1.5% FBS in 1 × DPBS for flow cytometry.

Frosted glass slides were used to dissociate spleens in 1X DPBS. Following dissociation, the cells were centrifuged at 4 °C for 5 min at 1500 rpm. Red blood cells were lysed with 1 mL of H_2_O, followed by the addition of 1 mL of 2 ×  DPBS, and the supernatant was transferred to a new conical tube for centrifugation. The splenocytes were resuspended with 1.5% FBS in 1 × DPBS for flow cytometry.

Flow cytometry was performed using a Becton Dickenson LSR II SORP (405/488/561/640) (BD Biosciences, San Jose, CA) and data analysis was carried out using FCS Express (RRID:SCR_016431; v.7.12; De Novo Software, Pasadena, CA; https://denovosoftware.com/). For macrophage analysis, the following antibody panel was used: FRβ-eFluor660-APC (Biolegend; 153305); F4/80-APC-R700 (BD-Horizon; 565787); CD80-PE-Cy5 (Invitrogen; 15-0801-81); ghost viability dye Violet 510 (TONBO biosciences, 13-0870-T100); BV605-conjugated CD45 (clone 30-F11, BD Biosciences, 563053CD11b-PE-CF594, BD Horizon; 562317); and Arg1-PE-Cy7 (Invitrogen; 25-3697-82).

For analysis of T cell populations, the following antibody panel was used: BV605-conjugated CD45 (clone 30-F11, BD Biosciences, 563053); APC efluor 780-conjugated CD3 (clone 17A2, Invitrogen, 47-0032-82); PerCP-Vio700-conjugated CD8α (clone 53–6.7, BD Bioscenices, 566410); Alexa Fluor-488-conjugated CD4 (clone RM4–5, Biolegend, 100532); and ghost viability dye Violet 510 (TONBO biosciences, 13-0870-T100).

Infiltrating macrophages were gated based on viable CD45+/CD11b+/F4/80 cells. M2-like FRβ positive macrophages were described as CD45+/CD11b+/F4/80+/Arg1+/FRβ. M1-like FRβ positive macrophages were described as CD45+/CD11b+/F4/80+/CD80+/FRβ. Infiltrating T cells were gated based on viable CD45+/CD3+ cells^31,32^. Infiltrating CD4+ and CD8+ T cells were gated based on viable CD45+/CD3+/CD4+ or CD8+ cells. The percentage of infiltrating CD3+, CD4+, CD8+, total macrophages, total FRβ macrophages, M1-like macrophages and M2-like macrophages were compared between control mice and **AGF94**-treated mice.

### Immunofluorescence

For immunofluorescence staining, harvested tumors were formalin fixed, embedded in paraffin, and tissue slides were cut at a thickness of 4 microns. Slides were deparaffinized with xylenes and subjected to a series of alcohol washes (100%, 95%, 70%), followed by antigen retrieval using a decloaking chamber (Biocare Medical) with 1X citrate. Slides were blocked for endogenous peroxidase with 3% H_2_O_2_ and treated for 1 h with 2.5% goat serum (Vector Labs, Catalog: S-1000). Primary antibodies, folate receptor β (FRβ) (1:250, Genetex, Catalog: GTX105822) and pan-macrophage F4/80, conjugated to APC-R700 (1:100, BD Biosciences, Catalog: 565787) were incubated overnight at 4 °C. The slides were incubated with anti-rabbit secondary antibody conjugated with Alexa Fluor 488 (1:200, ThermoFisher, Catalog: A-11008). Slides were mounted with Prolong Gold with 4,6-diamidino-2-phenylindole (DAPI) (Cell Signaling, Catalog: 9071) and images were acquired using confocal microscopy (LSM 780) at 40 × oil magnification. Images were corrected for brightness using Zen lite Blue software (https://www.zeiss.com/microscopy/us/products/microscope-software/zen-lite.html). Antibodies for CD3 (1:100, PT buffer, Cell Signaling, catalog#: 99940) and CD8 (1:50, Citrate, Cell Signaling, catalog#: #98941) were used with goat anti-rabbit IgG (H + L) Alexa Fluor 647. Slide processing and data acquisition are described above.

### Western blots of FRβ-expressing Chinese hamster ovary (CHO) cells with FRβ antibody

MTXRIIOua^R^2-4 (RFC-, PCFT- and FRα-null Chinese hamster ovary) cells (R2) were a gift from Dr. Wayne Flintoff (University of Western Ontario)^52^. Isogenic CHO cell lines were subsequently derived from R2 cells by transfection with FRα (RT16 cells) or FRβ (D4 cells) cDNAs^26^. The CHO sublines were grown in α-minimal essential medium (α-MEM) supplemented with 100 units/mL penicillin/100 μg/mL streptomycin, 2 mM *L*-glutamine and 10% bovine calf serum (Sigma-Aldrich).

For western blots, cells (~ 5 × 10^7^) were disrupted by sonication and cell debris removed by centrifugation (1,800 rpm, 5 min). A particulate membrane fraction was prepared by centrifugation at 37,000 × *g*. The membrane pellet was solubilized with 1% SDS in 10 mM Tris–HCl, pH 7 (containing protease inhibitors; Roche Diagnostics). Membrane proteins (28 μg) were electrophoresed on 10% Tris/glycine gels with SDS^53^ and transferred to polyvinylidene difluoride membranes (Thermo Scientific, Rockford, IL)^54^. The membrane was probed with FRβ antibody (1:500, Genetex, Catalog: GTX105822); detection was with IRDye700CW-conjugated goat anti-rabbit IgG secondary antibody (LI-COR Biosciences, Lincoln, NE). Membranes were scanned with an Odyssey® infrared imaging system (LI-COR Biosciences, Omaha, NE). Protein loading was normalized to levels of β-actin using a β-actin mouse antibody (Sigma-Aldrich).

### Statistical analyses

All data reflect at least three biological replicates unless noted otherwise. For in vitro cell-based assays, expression levels were assessed using unpaired t-test after log_2_ transformation to meet the normality assumption. For transcript analysis, a nonparametric Wilcoxon rank sum test was performed. For survival curves between control and **AGF94**-treated mice with IP BR-Luc tumors, a log-rank test was performed. For flow cytometry, a nonparametric Wilcoxon rank-sum test was performed. Statistical analyses were performed using Excel and Graphpad Prism 6.0.

## Supplementary Information

Supplementary Information.
