# Supplementary material for: Targeted therapy of pyrrolo[2,3-d]pyrimidine antifolates in a syngeneic mouse model of high grade serous ovarian cancer and the impact on the tumor microenvironment
Source: Sci Rep. 2022 Jul 5;12:11346. doi: 10.1038/s41598-022-14788-5 (PMC9256750; doi:10.1038/s41598-022-14788-5)
Supplement: Supplementary file 1 — Supplementary Information. [file 41598_2022_14788_MOESM1_ESM.pdf]

SUPPLEMENTARY MATERIALS

Targeted therapy of pyrrolo[2,3-*d*]pyrimidine antifolates in a syngeneic mouse model of high grade serous ovarian cancer and the impact on the tumor microenvironment

Adrianne Wallace-Povirk[1], Lisa Rubinsak[1], Agnes Malysa[1], Sijana H. Dzinic[1, 4], Manasa Ravindra[5], Mathew Schneider[1], James Glassbrook[3], Carrie O'Connor[1], Zhanjun Hou[1,4], Seongho Kim[1,4], Jessica Back[1, 4], Lisa Polin[1, 4], Robert T. Morris[1,4], Aleem Gangjee[5*], Heather Gibson[1,4*], and Larry H. Matherly[1,2,4*]

From the [1]Departments of Oncology, [2]Pharmacology, and [3]Biochemistry, Microbiology and Immunology, Wayne State University School of Medicine, and the [4]Barbara Ann Karmanos Cancer Institute, Detroit, Michigan; and the [5]Division of Medicinal Chemistry, Graduate School of Pharmaceutical Sciences, Duquesne University, Pittsburgh, Pennsylvania

Supplementary Table S1: Epithelial ovarian cancer (EOC) tissue cDNA array for real-time RT-PCR The cDNA array (Hort502) (Origene, Inc) contained 48 samples, including 8 normal, 8 stage I, 9 stage II, 17 stage III, and 6 stage IV EOC. Age, pathology, diagnosis, tumor grade and stage for each patient are summarized below.

Specimen	Age	Pathology	Diagnosis	Tumor grade	Stage
1	46	Normal	Leiomyoma of myometrium	Not Applicable	0
2	49	Normal	Leiomyoma of myometrium	Not Applicable	0
3	46	Normal	Leiomyoma of myometrium	Not Applicable	0
4	42	Normal	Endometrium, secretory	Not Applicable	0
5	33	Normal	No residual malignancy	Not Applicable	0
6	40	Normal	Cyst of ovary, follicular	Not Applicable	0
7	45	Normal	Endometriosis	Not Applicable	0
8	35	Normal	Endometriosis	Not Applicable	0
9	70	Tumor	Carcinoma of ovary, papillary serous	FIGO G2: Poorly differentiated	IA
10	68	Tumor	Adenocarcinoma of ovary, papillary cell	FIGO G2: Moderately differentiated	IA
11	48	Tumor	Adenocarcinoma of ovary, clear cell	Not Reported	IA
12	58	Tumor	Adenocarcinoma of ovary, endometrioid	FIGO G1: Well differentiated	IB
13	55	Tumor	Adenocarcinoma of ovary, endometrioid	FIGO G3: Poorly differentiated	IB
14	74	Tumor	Adenocarcinoma of ovary, endometroid, papillary serous	FIGO G3: Poorly differentiated	IB
15	65	Tumor	Carcinoma of ovary, papillary serous	FIGO G3: Poorly differentiated	IC
16	58	Tumor	Carcinoma of ovary, endometrioid	FIGO G2: Moderately differentiated	IC
17	78	Tumor	Adenocarcinoma of ovary, papillary serous	FIGO G2: Moderately differentiated	IIA
18	44	Tumor	Carcinoma of ovary, papillary serous	FIGO G2: Moderately differentiated	IIA
19	73	Tumor	Adenocarcinoma of ovary	FIGO G3: Poorly differentiated	IIA
20	67	Tumor	Adenocarcinoma of ovary, serous	FIGO G2: Moderately differentiated	IIB
21	67	Tumor	Adenocarcinoma of ovary, serous	FIGO G2: Moderately differentiated	IIB
22	58	Tumor	Adenocarcinoma of ovary, endometrioid	FIGO G3: Poorly differentiated	IIB
23	58	Tumor	Adenocarcinoma of ovary, papillary serous	FIGO G2: Moderately differentiated	IIB
24	59	Tumor	Adenocarcinoma of ovary, papillary serous	FIGO G3: Poorly differentiated	IIC
25	63	Tumor	Adenocarcinoma of ovary, serous	FIGO G2: Moderately differentiated	IIC
26	51	Tumor	Adenocarcinoma of ovary, serous	FIGO G2: Moderately differentiated	III
27	73	Tumor	Adenocarcinoma of ovary, serous	FIGO G3: Poorly differentiated	III
28	91	Tumor	Adenocarcinoma of ovary, papillary serous	FIGO G3: Poorly differentiated	III

29	75	Tumor	Adenocarcinoma of ovary, endometrioid	FIGO G3: Poorly differentiated	IIIA
30	65	Tumor	Adenocarcinoma of ovary, serous	FIGO G3: Poorly differentiated	IIIA
31	55	Tumor	Adenocarcinoma of ovary, mucinous	FIGO G2: Moderately differentiated	IIIA
32	66	Tumor	Adenocarcinoma of ovary, serous	FIGO G2: Moderately differentiated	IIIA
33	80	Tumor	Adenocarcinoma of ovary, serous	Not Reported	IIIB
34	64	Tumor	Adenocarcinoma of ovary, papillary serous	FIGO G3: Poorly differentiated	IIIB
35	66	Tumor	Carcinoma of ovary, papillary serous	FIGO G3: Poorly differentiated	IIIB
36	52	Tumor	Adenocarcinoma of ovary, papillary serous	FIGO G3: Poorly differentiated	IIIB
37	71	Tumor	Adenocarcinoma of ovary, papillary serous	FIGO G3: Poorly differentiated	IIIC
38	48	Tumor	Adenocarcinoma of ovary, papillary serous	FIGO G2: Moderately differentiated	IIIC
39	50	Tumor	Carcinoma of ovary, papillary serous	FIGO G3: Poorly differentiated	IIIC
40	58	Tumor	Adenocarcinoma of ovary, papillary serous	FIGO G2: Moderately differentiated	IIIC
41	53	Tumor	Adenocarcinoma of ovary, serous	FIGO G3: Poorly differentiated	IIIC
42	66	Tumor	Adenocarcinoma of ovary, endometrioid, papillary serous	FIGO G3: Poorly differentiated	IIIC
43	45	Tumor	Adenocarcinoma of ovary, serous, metastatic	Not Reported	IV
44	80	Tumor	Adenocarcinoma of ovary, endometroid, papillary serous	FIGO G3: Poorly differentiated	IV
45	68	Tumor	Adenocarcinoma of ovary, serous	FIGO G3: Poorly differentiated	IV
46	61	Tumor	Adenocarcinoma of ovary, serous	FIGO G3: Poorly differentiated	IV
47	60	Tumor	Adenocarcinoma of ovary, papillary serous, metastatic	Not Reported	IV
48	63	Tumor	Adenocarcinoma of ovary, papillary serous	FIGO G3: Poorly differentiated	IV

Supplementary Table S2. Primer sequences for C1 genes and housekeeping genes for murine (“m”) and human cells.

Gene	Forward Sequence	Reverse Sequence
**Mouse**		
mAICARFTase	cccaaacttcccatcacagt	cgttcaaagcgtcacacaag
mGARFTase	acattggcttccgtgctg	tccggagtccttgtaagtcaa
mFR	ctcctggtgtgtcacaggatt	ggcttcctttgctttcttca
mRFC	gggtctccaagtccgtgat	ccacgctggcagtacctta
mPCFT	tacccggccactctgaact	tccgggtgtggattaacct
mβ-actin	ctaaggccaaccgtgaaaag	accagaggcatacagggaca
**Human**		
β-actin	caaccgcgagaagatgac	gtccatcacgatgccagt
GARFTase	ccctgagaaacttggggtaga	ctgctgcaaccatgagaaaa
AICARFTase	gagggactgcaaaagctctc	ggaaatcccgtcaactcaga

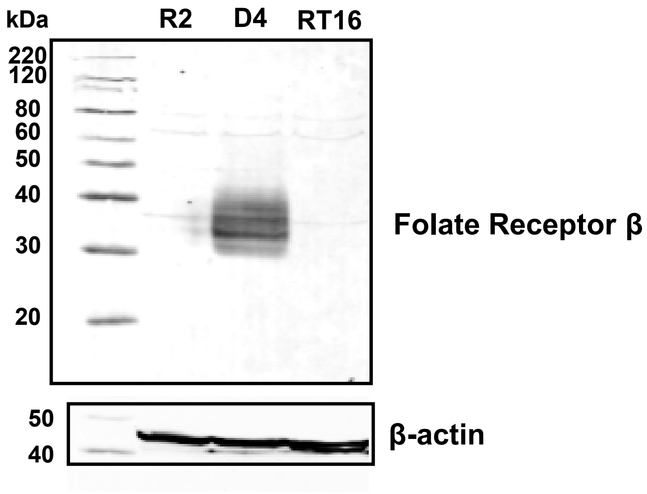

Figure S1. Folate Receptor (FR) β specificity by western blot of FRβ-expressing Chinese hamster ovary cells. Chinese hamster ovary cells null for FRs (R2) or expressing FRβ (D4) or FRα (RT16) were measured from a particulate membrane fraction by SDS polyacrylamide gel electrophoresis and Western blotting probed with FRβ-specific antibody (Genetex, Catalog: GTX105822); detection was with IRDye700CW-conjugated goat anti-rabbit IgG secondary antibody (LI-COR Biosciences, Lincoln, NE). Membranes were scanned with an Odyssey® infrared imaging system (LI-COR Biosciences, Omaha, NE). Protein loading was normalized to levels of β-actin using anti-β-actin mouse antibody (Sigma-Aldrich).

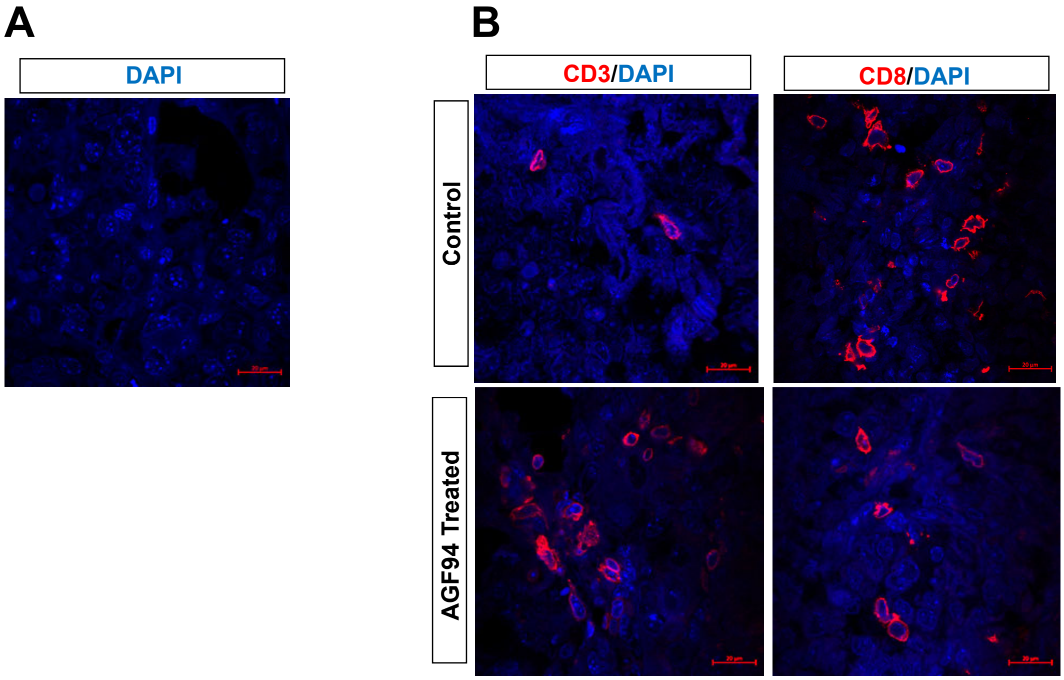

Figure S2. Negative control for immunofluorescence and immunofluorescence staining for CD3 and CD8. Panel A. Control immunofluorescence staining with secondary antibody (anti-Rabbit IgG (H+L) Alexa Fluor 647 (1:200, Thermo Fisher, Catalog# A-21245). Panel B. Immunofluorescence staining for CD3 (Cell Signaling) and CD8 (Cell Signaling) with goat anti-rabbit IgG (H+L) Alexa Fluor 647 as described in the Materials and Methods. Scale bar set to 20 µm.
